# The relationship between coronary artery distensibility and fractional flow reserve

**DOI:** 10.1371/journal.pone.0181824

**Published:** 2017-07-25

**Authors:** Andy S. C. Yong, Ashkan Javadzadegan, William F. Fearon, Abouzar Moshfegh, Jerrett K. Lau, Stephen Nicholls, Martin K. C. Ng, Leonard Kritharides

**Affiliations:** 1 Faculty of Medicine and Health Sciences, Macquarie University, Sydney, New South Wales, Australia; 2 ANZAC Research Institute, The University of Sydney, Sydney, New South Wales, Australia; 3 Department of Cardiology, Concord Hospital, The University of Sydney, Sydney, New South Wales, Australia; 4 Division of Cardiovascular Medicine, Stanford University Medical Center, Stanford, California, United States of America; 5 South Australian Health and Medical Research Institute, Adelaide, South Australia, Australia; 6 Royal Prince Alfred Hospital, The University of Sydney, Sydney, New South Wales, Australia; Medstar Washington Hospital Center, UNITED STATES

## Abstract

Discordance between angiography-based anatomical assessment of coronary stenosis severity and fractional flow reserve (FFR) has been attributed to several factors including lesion length and irregularity, and the myocardial territory supplied by the target vessel. We sought to examine if coronary arterial distensibility is an independent contributor to this discordance. There were two parts to this study. The first consisted of “in silico” models of 26 human coronary arteries. Computational fluid dynamics-derived FFR was calculated for fully rigid, partially distensible and fully distensible models of the 26 arteries. The second part of the study consisted of 104 patients who underwent coronary angiography and FFR measurement. Distensibility at the lesion site (Distensibility_MLA_) and for the reference vessel (Distensibility_Ref_) was determined by analysing three-dimensional angiography images during end-systole and end-diastole. Computational fluid dynamics-derived FFR was 0.67±0.19, 0.70±0.18 and 0.75±0.17 (P<0.001) in the fully rigid, partially distensible and fully distensible models respectively. FFR correlated with both Distensibility_MLA_ (r = 0.36, P<0.001) and Distensibility_Ref_ (r = 0.44, P<0.001). Two-way ANCOVA analysis revealed that Distensibility_MLA_ (F (1, 100) = 4.17, p = 0.031) and percentage diameter stenosis (F (1, 100) = 60.30, p < 0.01) were both independent predictors of FFR. Coronary arterial distensibility is a novel, independent determinant of FFR, and an important factor contributing to the discordance between anatomical and functional assessment of stenosis severity.

## Introduction

Fractional flow reserve (FFR) is a well-accepted method to assess the functional significance of coronary stenosis in the cardiac catheterization laboratory. Use of FFR to guide revascularization decisions leads to improved outcomes [1, 2]. Several studies have shown reasons for the discordance between FFR and anatomical methods to assess lesion severity, which include lesion irregularity, lesion length, the presence of diffuse coronary artery disease as well as the size and status of the microcirculation supplied by the target vessel [3, 4]. Vessel wall distensibility could be another factor that affects FFR. Increased distensibility will likely lead to decreased resistance to flow and lower pressure gradient across a particular stenosis. Therefore, assuming two vessels with identical geometry and stenosis severity, the vessel with greater distensibility will likely have a higher FFR value. There is a lack of studies that examine whether vessel wall distensibility contributes to this discordance. We hypothesize that increased vessel wall distensibility will be associated with higher FFR values. In the current study, we aim to investigate whether coronary arterial distensibility will affect the discordance between angiography-based anatomical assessment of stenosis severity and FFR.

Lower FFR, when unrevascularized, is associated with adverse outcomes [5–6]. Factors that may cause stiffness or low distensibility such as calcification have been associated with poor prognosis [7]. The association between distensibility and FFR may therefore also provide a mechanistic link to explain the ability of FFR to predict adverse outcome.

Several new techniques have emerged to indirectly calculate FFR. Computational fluid dynamics (CFD) is the use of applied mathematics and physics incorporated in computer software to characterise how a gas or liquid flows. The use of CFD-based techniques has led to less invasive, and potentially clinically applicable methods to derive FFR. Computed tomography (CT) coronary angiography and three-dimensional coronary angiography can both render accurate anatomic reconstruction of coronary artery geometry. Using CFD, coronary angiography-derived FFR, which does not require the pressure-sensor wire, and CT-derived FFR, which is a non-invasive method have been developed [8–14]. These CFD-based techniques have incorporated known factors that cause the discordance between anatomical and functional methods to assess lesion severity including lesion irregularity, lesion length, the presence of diffuse coronary artery disease as well as the size and status of the microcirculation supplied by the target vessel [11, 14], but remain prone to inaccurate calculation of FFR [14, 15]. All CFD-derived FFR methods to date have used static vessel geometry. Incorporating distensibility may improve the accuracy of CFD methods to predict FFR.

## Methods

There were two parts to this study. The first consisted of a CFD simulation study in a group of coronary artery geometries with different grades of stenosis severity to determine whether vessel wall distensibility directly affects FFR in an “in silico” model. The second part consisted of a clinical study of patients who underwent FFR measurements. The relationship between vessel wall distensibility and FFR was investigated in these patients. This study was approved by the Sydney Local Health District Human Research Ethics Committee-Concord Repatriation General Hospital with additional site specific approval by the Research Ethics and Governance Office of the Royal Prince Alfred Hospital. Written informed consent was obtained from patients who participated in this study and the study protocol conforms to the ethical guidelines of the 1975 Declaration of Helsinki as reflected in a priori approval by the institution's human research committee. All angiographic and CFD analyses were performed at the coronary CFD core laboratory at the ANZAC Research Institute.

### Simulation study

#### Coronary geometries

We selectively identified 30 consecutive coronary angiograms of patients who had FFR measurement in a single vessel at our institution. We consecutively identified 10 vessels with low grade stenoses (<40%DS), 10 vessels with intermediate grade stenoses (40–70%DS) and 10 vessels with high-grade stenoses (>70%DS). Of these 30 vessels, 26 were found to have adequate image quality for three-dimensional reconstruction.

#### Three-dimensional coronary vessel reconstruction

**Fig 1:**
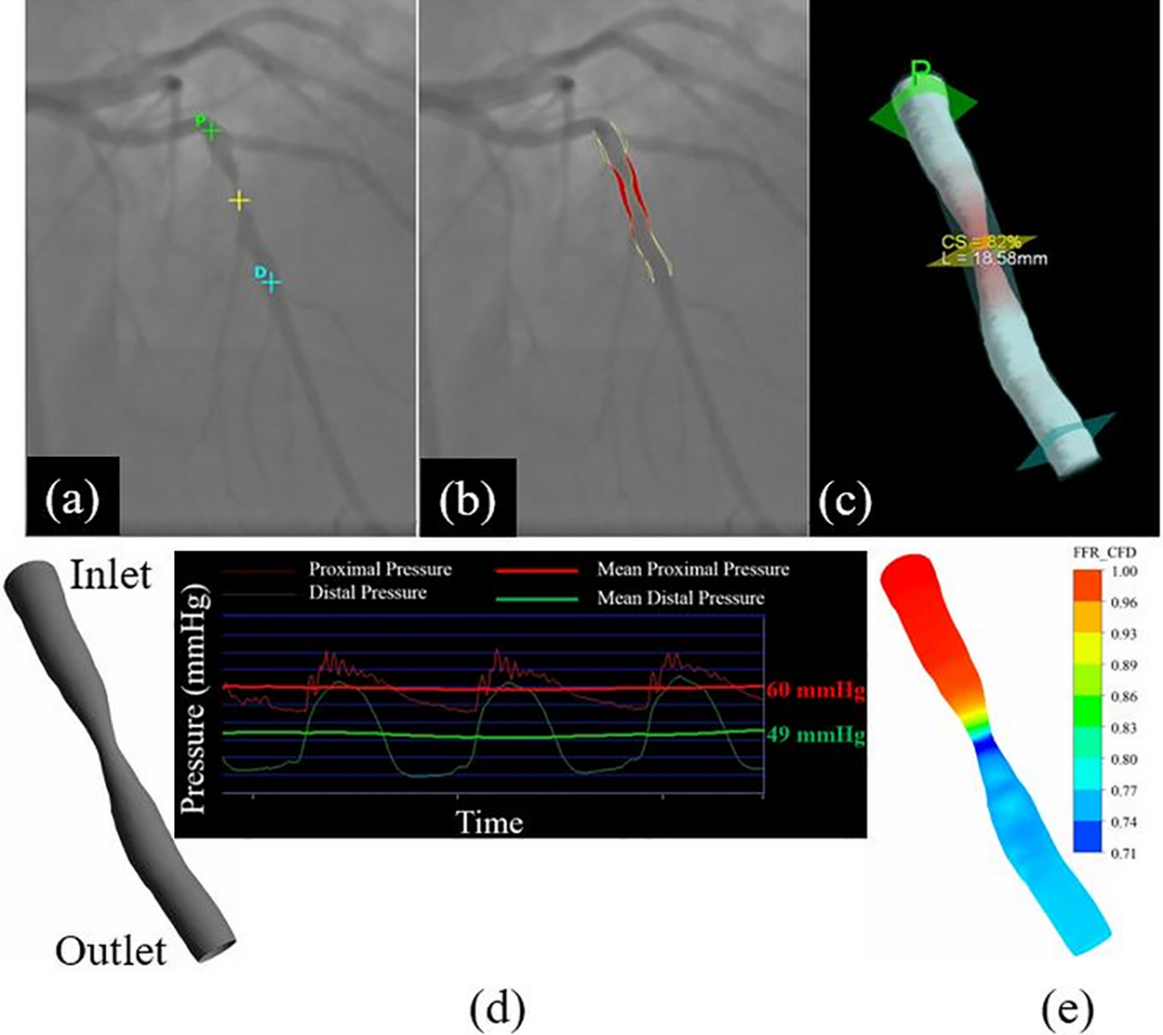
CFD model. (a) Coronary angiogram of LAD artery with initial definition of lesion, and non-stenotic segments proximal and distal to the lesion, (b) Two-dimensional representation of vessel lumen, (c) Three-dimensional reconstruction of vessel lumen showing lesion length (L) and percentage cross sectional area stenosis (CS). Yellow cross defines lesion site, cross denoted by “P” defines proximal site and cross denoted by “D” defines distal site, (d) Defined inlet and outlet with the corresponding boundary condition, (e) contours of CFD-based FFR.

Three-dimensional quantitative coronary angiography (3D-QCA) was employed to reconstruct the coronary luminal geometry using previously described methods [16]. In brief, angiographic cine images were acquired at 15 frames per second, consistent with the protocol at our institution (Axiom Artis, Siemens, Forchheim, Germany). After inspection of images, locations of narrowing as well as proximal and distal segments of each coronary artery were manually identified. Subsequently the centre line of the arterial lumen was manually identified (Fig 1a and 1b). Finally, the vessels were reconstructed offline using 3DR software on the Leonardo workstation (IC3D, Siemens, Forchheim, Germany) (Fig 1c). The contrast-filled non-tapered part of the guiding catheter was used to calibrate pixel size. The two best orthogonal angiographic views of the vessel in the end-diastolic frame were used for reconstruction.

#### CFD computed FFR in models with different vessel distensibility

ANSYS 14.5 (ANSYS, Inc., Canonsburg, PA, USA) was used for CFD simulations. Flow for modelling was assumed to be three-dimensional and Newtonian. It has previously been shown that flow in stenosed coronary arteries may transit to turbulence [17]. In this study, shear stress transport turbulent model was used to capture the transition, if any, to a turbulent state [18, 19].

First, steady state simulations were conducted with rigid wall assumption as previously published [20], to calculate the patient-specific hyperemic flow rate. In brief, the measured proximal and distal pressures during hyperemia were used respectively as inlet and outlet boundary conditions (Fig 1d) and patient-specific CFD-based hyperemic flow rate was derived. Then, semi-transient fluid—structure interaction (FSI) simulations [21] (Fig 1e) were conducted with elastic wall assumption with three grades of distensibility; perfectly distensible (ART_distensible_), partially distensible (ART_partial_) and non-distensible (ART_rigid_). In the perfectly distensible model, the vessel walls were allowed to dilate freely and gradually from end diastole to end systole, based upon the measured patient-specific distensibility, over a number of simulation time steps. To simulate semi-transient flow, repeated simulations were performed to include up to 100 steady state iterations. In the partially distensible model, the vessel walls were constrained such that the distensibility was brought down to half of the measured patient-specific distensibility. In the non-distensible model, the vessel walls were fully constrained to prevent dilation.

For the FSI simulations, the inlet boundary condition was set to measured aortic pressure during angiography and the outlet boundary condition was initially set to the computed patient-specific hyperemic flow rate. During the simulation, proximal pressure was kept constant. The flow rate and outlet pressure were allowed to vary in order to keep microcirculation resistance constant.

Blood was modelled as an incompressible Newtonian fluid with dynamic viscosity of 0.0035 Pa.s and density of 1050 kg/m^3^ [16, 20]. To incorporate the elastic nature of coronary arterial walls, the conventional Mooney-Rivlin hyperelastic model was used [22].

CFD-based FFR (FFR_CFD_) was calculated by the ratio of hyperemic distal pressure to proximal pressure for the 3 distensibility models using all 26 vessels. The differences in FFR_CFD_ among the 3 different models were determined.

### Clinical study

#### Patient population

The study population consisted of 104 consecutive patients who presented to the cardiac catheterization laboratory and required FFR measurement of a single target lesion at two tertiary referral institutions. All patients underwent coronary angiography and physiological measurements.

#### Pressure and FFR measurements

FFR was measured using a pressure-sensor guidewire (Aeris or Certus pressure wire, St. Jude, MN, USA) as previously described [16]. The pressure-sensor wire was first calibrated and then equalized with the pressure measurement obtained from the guiding catheter. The wire was then advanced distal to the target lesion. Intracoronary nitroglycerin (100–200μg) was administered. Hyperemia was achieved using intravenous adenosine infusion at a rate of 140 μg/kg/min. Adenosine was infused for at least 2 minutes to obtain a stable hyperemic signal. FFR was calculated as the mean distal pressure divided by the mean proximal pressure during hyperemia. The pressure-sensor was pulled back after FFR measurement to check for pressure drift. Pressure equalization and FFR measurement was repeated if significant pressure drift was found.

#### Coronary vessel distensibility measurement

**Fig 2:**
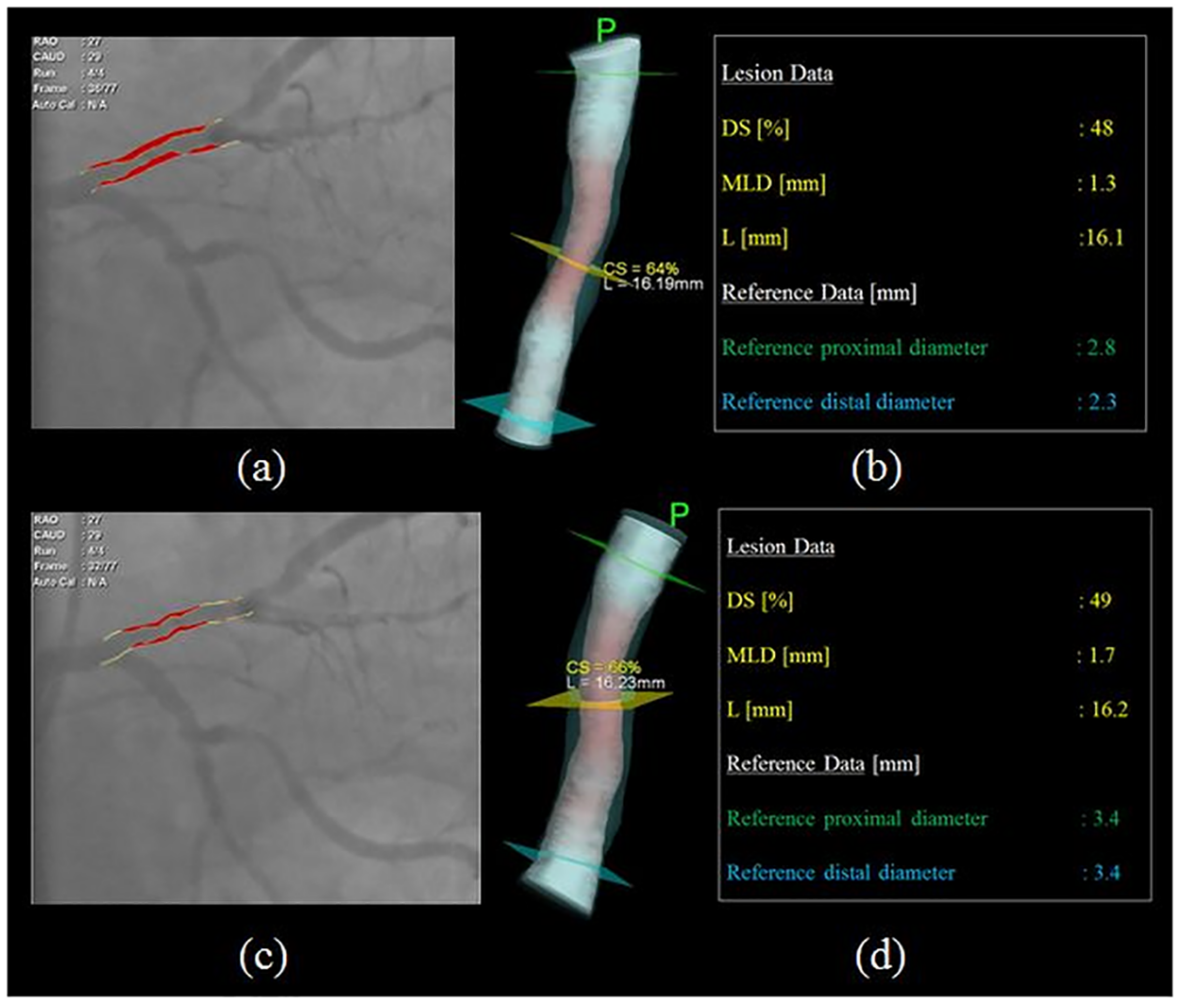
Representative three-dimensional reconstruction of vessel geometry. (a) End-diastolic two-dimensional representation of lesion, (b) End-diastolic three-dimensional reconstruction of vessel lumen with corresponding measurements, (c) End-systolic two-dimensional representation of lesion, (d) End-systolic three-dimensional reconstruction of vessel lumen with corresponding measurements. DS, MLD, and L represent diameter stenosis, minimum luminal diameter and lesion length.

Coronary artery geometries were obtained as described for the first part of the study. 3D-QCA was used to determine reference vessel size, %DS and lesion length. Coronary vessel distensibility calculations were performed by analysing 3D angiography images of coronary lesions during end-systole and end-diastole. We ensured that all selected images had adequate quality for calculating the distensibility and the frames at which the vessel was fully filled by contrast were selected for analysis. All measurements were performed thrice and averaged by a single-experienced operator blinded to the FFR results. Inter-observer error was determined by a second operator for a random selection of 20 vessels. Fig 2 illustrates 2D angiographic images and 3D reconstructed geometry of a representative left anterior descending artery at end-systole and end-diastole. The following formulae were used to quantify vessel distensibility at the site of minimum luminal area and for the proximal reference vessel as previously described [23, 24]:

$${\text{Distensibility}}_{\text{MLA}}=\;\frac{1000{\left(\Delta \text{A}\right)}_{\text{MLA }}/\;{\left({\text{A}}_{\text{d}}\right)}_{\text{MLA}}}{\Delta \text{p}}$$

$${\text{Distensibility}}_{\text{Ref}}=\;\frac{1000{\left(\Delta \text{A}\right)}_{\text{Ref }}/\;{\left({\text{A}}_{\text{d}}\right)}_{\text{Ref}}}{\Delta \text{p}}$$

(ΔA)_MLA_ and (ΔA)_Ref_ represent the difference between end-systolic and end-diastolic area at the site of minimum luminal area and proximal reference vessel respectively. (A_d_)_MLA_ and (A_d_)_Ref_ represent end-diastolic area at the site of minimum luminal area and proximal reference vessel respectively. Δp represents the difference between mean systolic and diastolic intracoronary pressure [25]. In order to validate the use of 3D-QCA to measure distensibility, we carried out a comparison of distensibility measured by intravascular ultrasound (IVUS) and distensibility measured by 3D-QCA in 20 patients who underwent IVUS in a single vessel.

### Statistical analysis

As this was a novel pilot study without prior clinical data for reference, we aimed to include 100 patients for the clinical study. Graphpad Prism v. 5.01 (Graphpad, La Jolla, California) and SPSS v. 15 (SPSS, Chicago, Illinois) software were used to perform statistical analyses. All the values were expressed as mean ± SD unless otherwise specified. Normality of the data was determined using the D′Agostino Pearson test and verified using histogram plots. Parametric and non-parametric statistical analyses were performed accordingly. T-tests were used to compare means between two groups, and one-way ANOVA analysis was used to compare means between greater than two groups of variables. Non-linear regression analysis was used to determine the relationship between %DS and change in FFR in different arterial stiffness models. Pearson correlation analyses were used to compare the association between two continuous variables. Two-way ANCOVA was used to determine whether distensibility was an independent predictor of FFR. Two-sided P value of < 0.05 was considered significant.

## Results

### Simulation study

**Fig 3:**
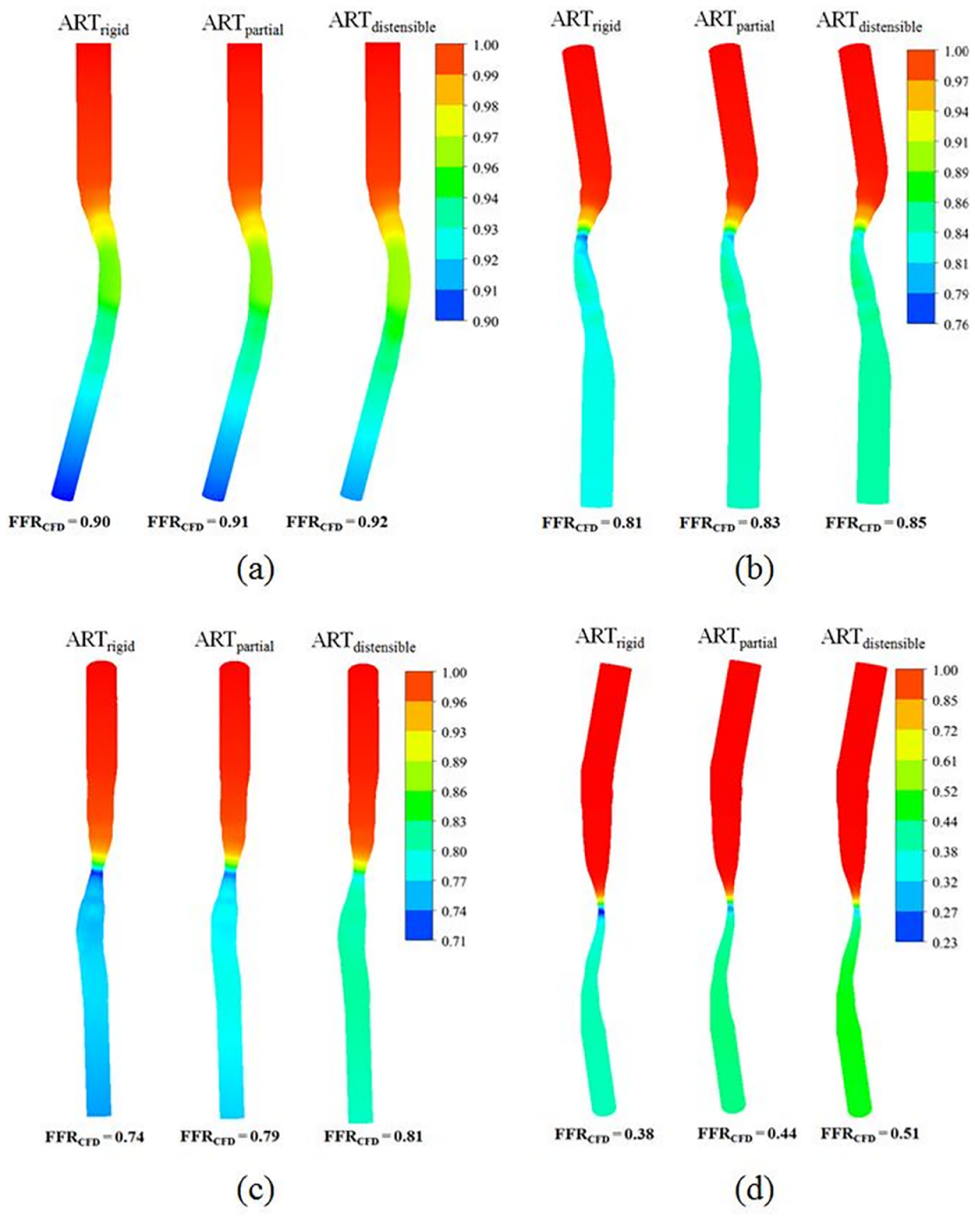
Effect of vessel rigidity on FFR_CFD_. FFR_CFD_ for representative arteries with percentage diameter stenosis of (a) 40, (b) 50.5, (c) 62.5, and (d) 73 are shown.

Fig 3 shows the contour plots of FFR_CFD_ in 4 representative coronary geometries with varying distensibility (ART_rigid_, ART_partial_ and ART_distensible_) and diameter stenosis. As shown, FFR_CFD_ was lower in the ART_rigid_ models when compared with the ART_partial_ and ART_distensible_ models. The difference in FFR_CFD_ between rigid and distensible models became greater with increasing stenosis severity.

**Fig 4:**
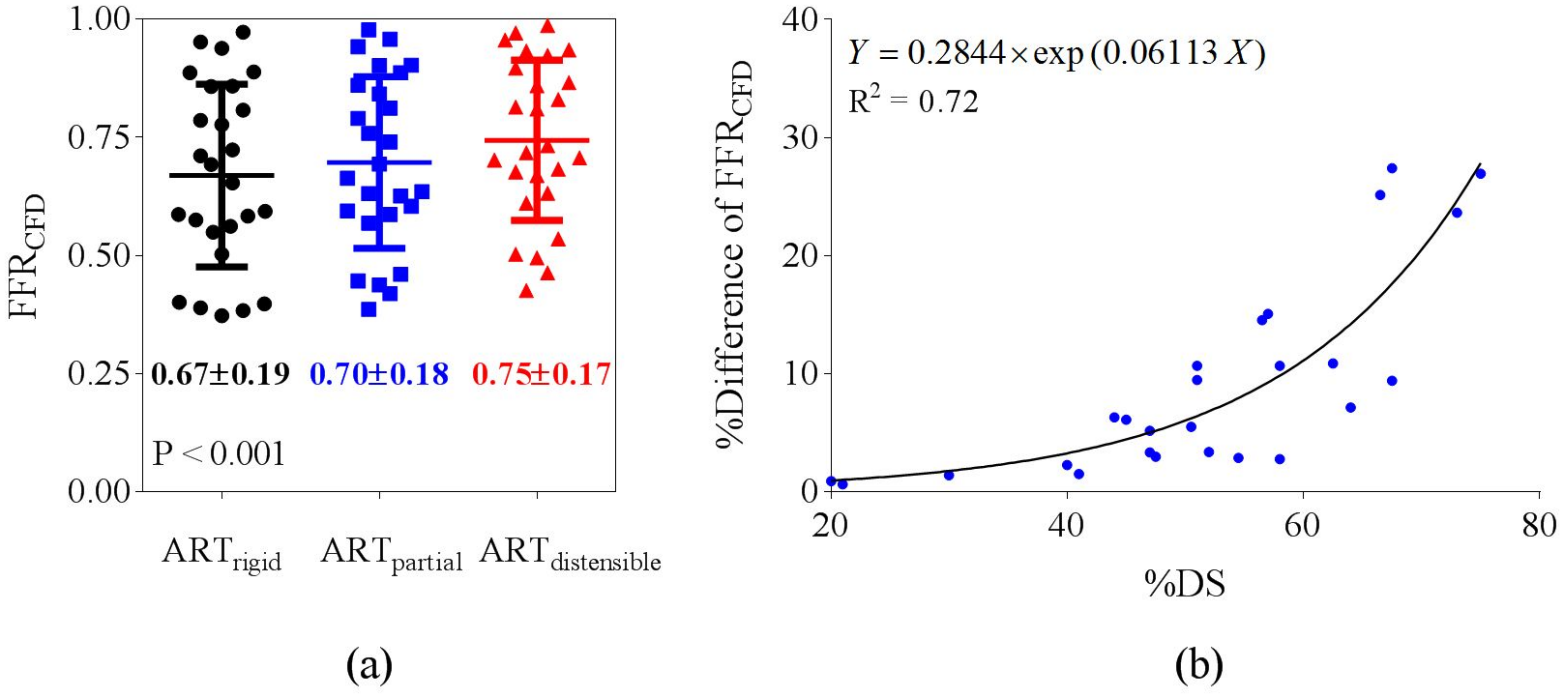
FFR_CFD_ decreases with increasing vessel rigidity. (a) FFR_CFD_ in ART_rigid_, ART_partial_ and ART_distensible_ models (P value was derived from one-way ANOVA analysis), (b) Non-linear regression curve showing relationship between percentage diameter stenosis and percentage difference in FFR_CFD_ between ART_distensible_ and ART_rigid_ models. $\%\text{Difference}\;\text{of}\;\text{FF}{\text{R}}_{\text{CFD}}\;=\;\;100\times \left[{\left(\text{FF}{\text{R}}_{\text{CFD}}\right)}_{\text{AR}{\text{T}}_{\text{distensible}}}-\;{\left(\text{FF}{\text{R}}_{\text{CFD}}\right)}_{\text{AR}{\text{T}}_{\text{rigid}}}\right]/{\left(\text{FF}{\text{R}}_{\text{CFD}}\right)}_{\text{AR}{\text{T}}_{\text{distensible}}}$

When all 26 coronary geometries were analyzed, FFR was 0.67±0.19, 0.70±0.18 and 0.75±0.17 (P<0.001) in the ART_rigid_, ART_partial_ and ART_distensible_ models respectively (Fig 4a). The difference in FFR_CFD_ between ART_rigid_ and ART_distensible_ models increased in an exponential manner with increasing stenosis severity (Fig 4b).

**Fig 5:**
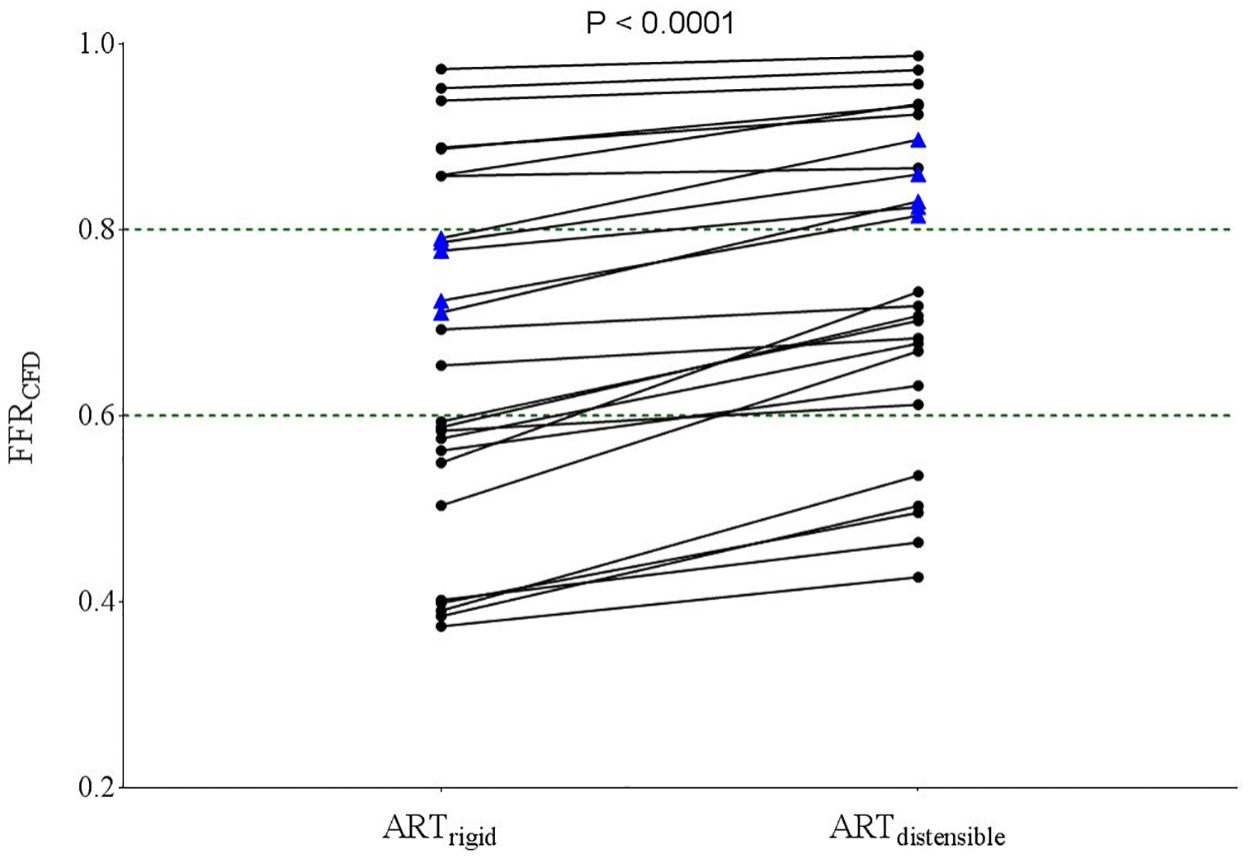
FFR_CFD_ in ART_rigid_ and ART_distensible_ models for 26 coronary arteries. Blue triangles represent the coronary arteries with FFR < 0.8 when modeled as rigid vessels and had FFR > 0.8 when modeled as distensible vessels.

In 5 out of the 26 (19.2%, shown by blue triangles) of the patient geometries, FFR was originally ≤0.8 when using a rigid model but became >0.8 when a fully distensible model was used. When considering patient geometries with rigid model FFR between 0.6–0.8 (shown by dashed lines), 5 out of the 7 (71.4%) had FFR >0.8 when a distensible model was used (Fig 5).

### Clinical study

Demographic, clinical and angiographic characteristics of the 104 patients in this study are shown in Table 1. Mean Distensibility_MLA_ was 2.95 ± 1.27 mmHg^-1^ and mean Distensibility_Ref_ was 3.83 ±1.45 mmHg^-1^. Absolute mean intra-observer error for Distensibility_MLA_ and Distensibility_Ref_ were 0.25 ± 0.26 mmHg^-1^ and 0.38 ± 0.35 mmHg^-1^ respectively. Absolute mean inter-observer error for Distensibility_MLA_ and Distensibility_Ref_ were 0.37 ± 0.27 mmHg^-1^ and 0.49 ± 0.13 mmHg^-1^ respectively.

**Table 1:** Baseline clinical and lesion characteristics.

Variable	n = 104
Age (years)	63 ± 9.9
Male sex, n (%)	82 (78.8)
Clinical history, n (%)	
Hypertension	66 (63.4)
Dyslipidaemia	76 (73.1)
Diabetes	37 (35.6)
History of smoking	43 (41.3)
Medications, n (%)	
Aspirin	101 (97.0)
Clopidogrel	77 (74.0)
Beta-blocker	62 (59.6)
ACE-I/ARB	57 (54.8)
Statin	87 (83.6)
Lesion characteristics	
Left anterior descending artery, n (%)	72 (69.5)
Left circumflex artery, n (%)	10 (9.5)
Right coronary artery, n (%)	22 (21)
Myocardial bridging, n (%)	2 (1.9)
Reference vessel size (mm)	2.8 ± 0.5

Values represent mean ± SD.

ACE-I, angiotensin-converting enzyme inhibitor; ARB, angiotensin receptor blocker.

**Fig 6:**
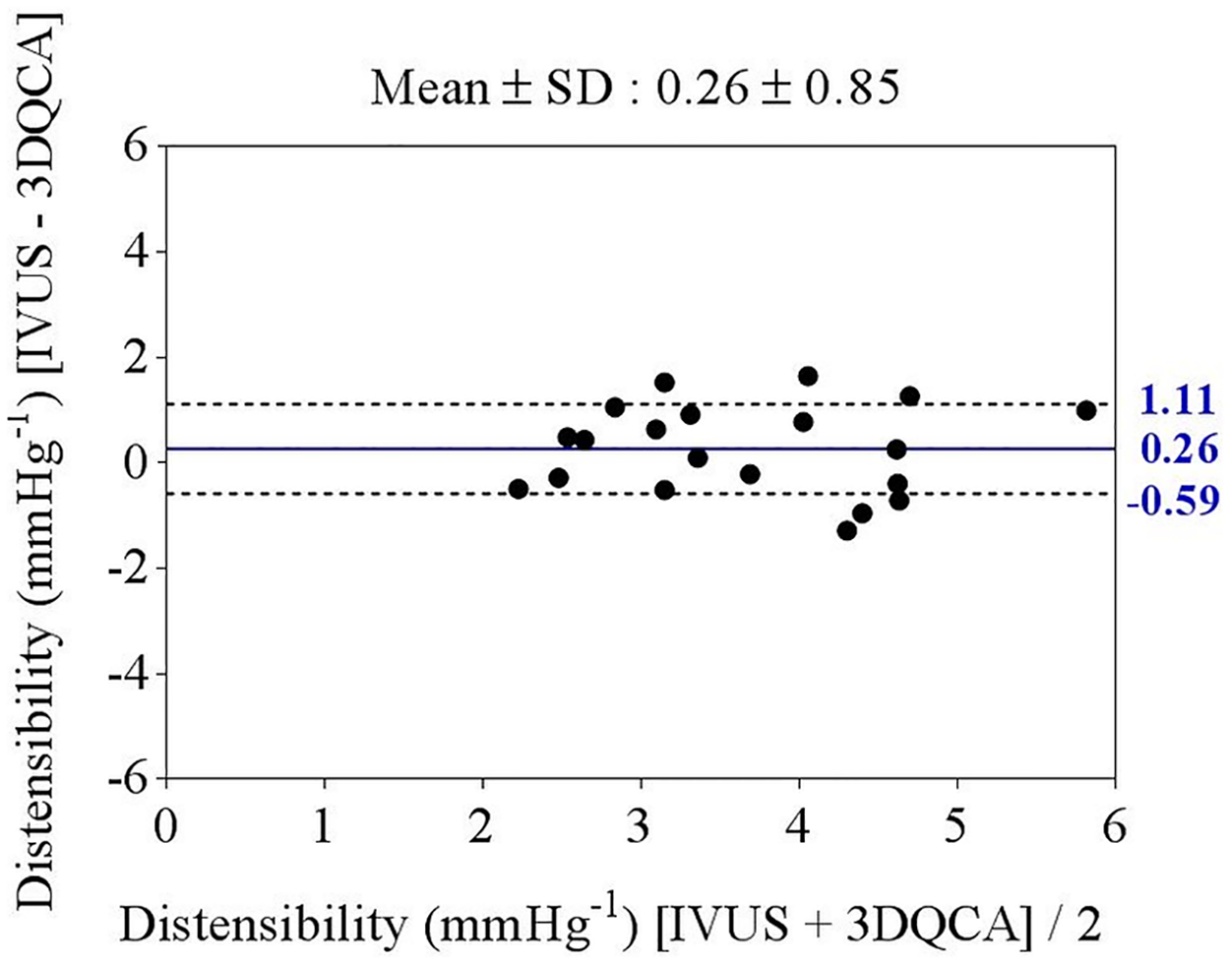
Bland-Altman analysis of the comparison between IVUS and 3D-QCA derived distensibility.

In a subgroup of 20 CAD patients, the luminal area as well as distensibility measurements between IVUS and 3D-QCA were compared. The reference luminal area during end diastole was 8.2 ± 0.6 using 3D-QCA and 9.2 ± 0.7 using IVUS (P = 0.31). The reference luminal area during end systole was 9.5 ± 0.7 using 3D-QCA and 10.9 ± 0.7 using IVUS (P = 0.22). There was no significant difference between IVUS and 3D-QCA derived distensibility (3.81 ± 1.04 mmHg^-1^ versus 3.55 ± 1.05 mmHg^-1^, P = 0.44), and there was good agreement between the two methods (Fig 6).

Both Distensibility_MLA_ and Distensibility_Ref_ correlated inversely with age, and there was a non-significant trend for patients with hypertension to have lower Distensibility_MLA_ compared to patients without hypertension (S1 Table). There was no significant relationship between distensibility and other clinical variables including medication usage (S1 Table). There was no significant relationship between FFR and any of the clinical variables including medication usage (S2 Table).

**Fig 7:**
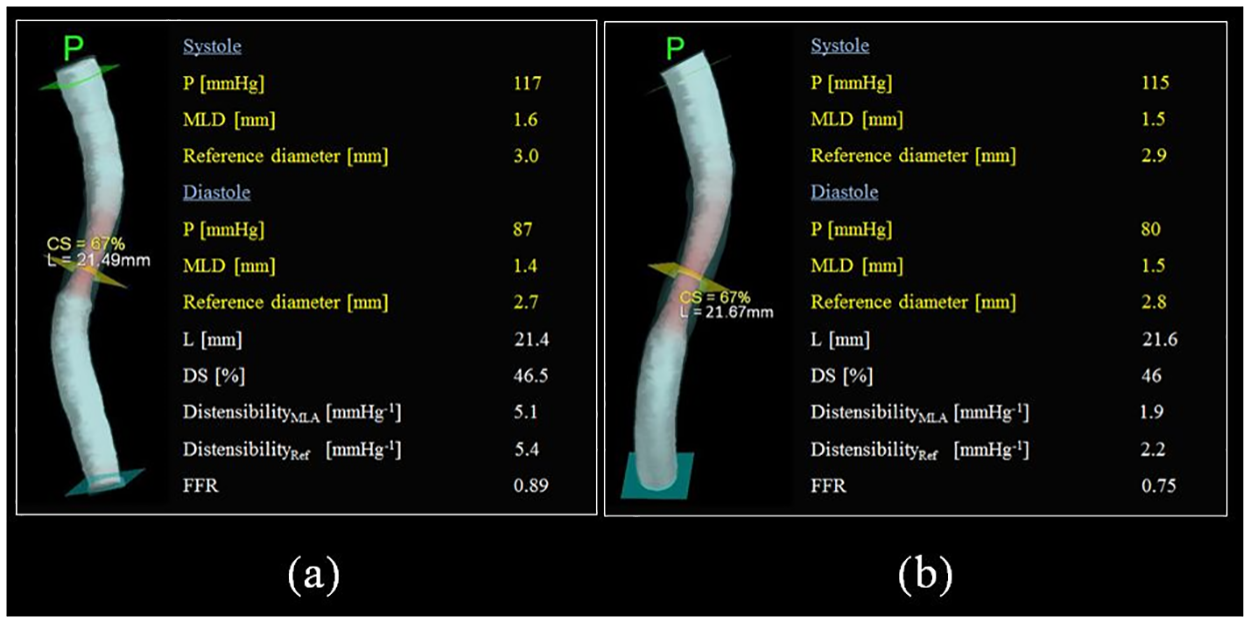
Representative comparison of two coronary arteries with similar lesion characteristics but different distensibility. (a) vessel with high distensibility, (b) vessel with low distensibility.

Fig 7 shows two representative coronary arteries with very similar lesion and reference vessel characteristics (%DS, minimum luminal diameter and reference vessel size). As shown, despite matched stenosis severity, the coronary vessel with higher distensibility had FFR of 0.89 whereas the less distensible vessel had FFR of 0.75.

**Fig 8:**
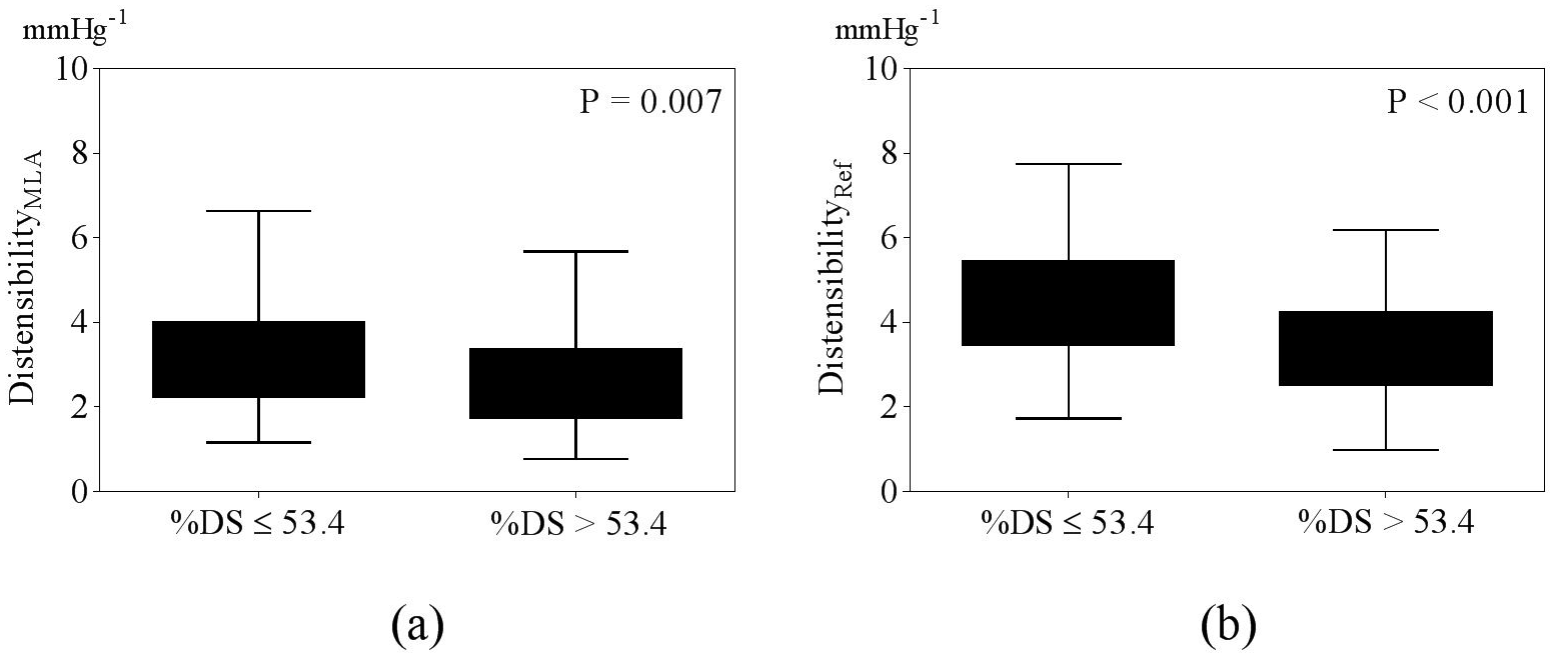
Relationship between stenosis severity and distensibility. (a) Distensibility_MLA_ and (b) Distensibility_Ref_.

Mean %DS was 53.4 ± 13.6% and mean lesion length was 12.2 ± 6.4 mm. Both Distensibility_MLA_ and Distensibility_Ref_ were significantly higher in coronary arteries with %DS ≤ 53.4 compared to arteries with %DS > 53.4 (Fig 8). As expected, FFR correlated with %DS (r = -0.66, P < 0.001) and lesion length (r = -0.24, P = 0.01). Lesion length correlated with Distensibility_Ref_ (r = -0.23, P = 0.02) but not Distensibility_MLA_ (r = -0.17, P = 0.09).

**Fig 9:**
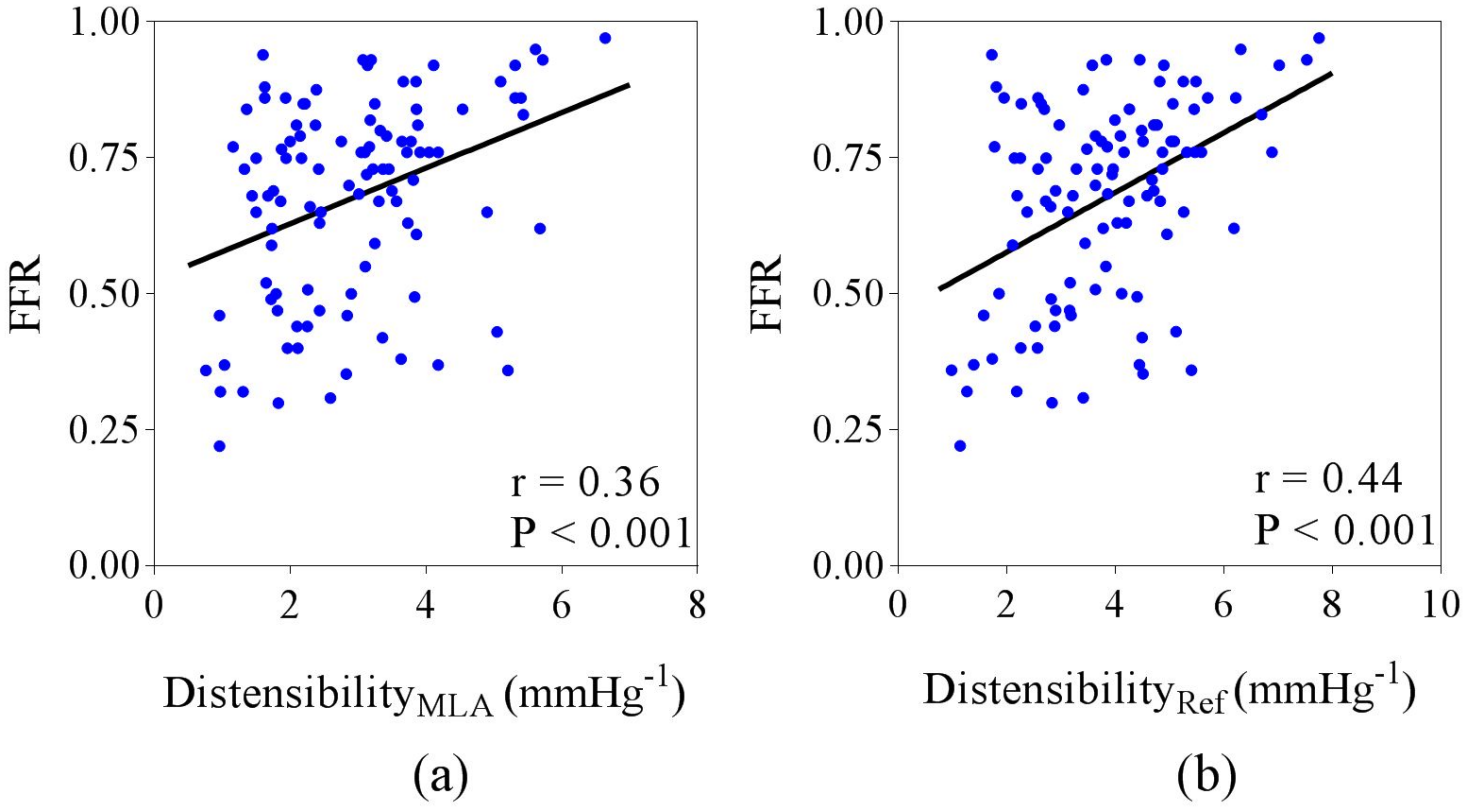
Scatterplots showing relationship between FFR and distensibility. (a) Distensibility_MLA_ and (b) Distensibility_Ref_.

**Fig 10:**
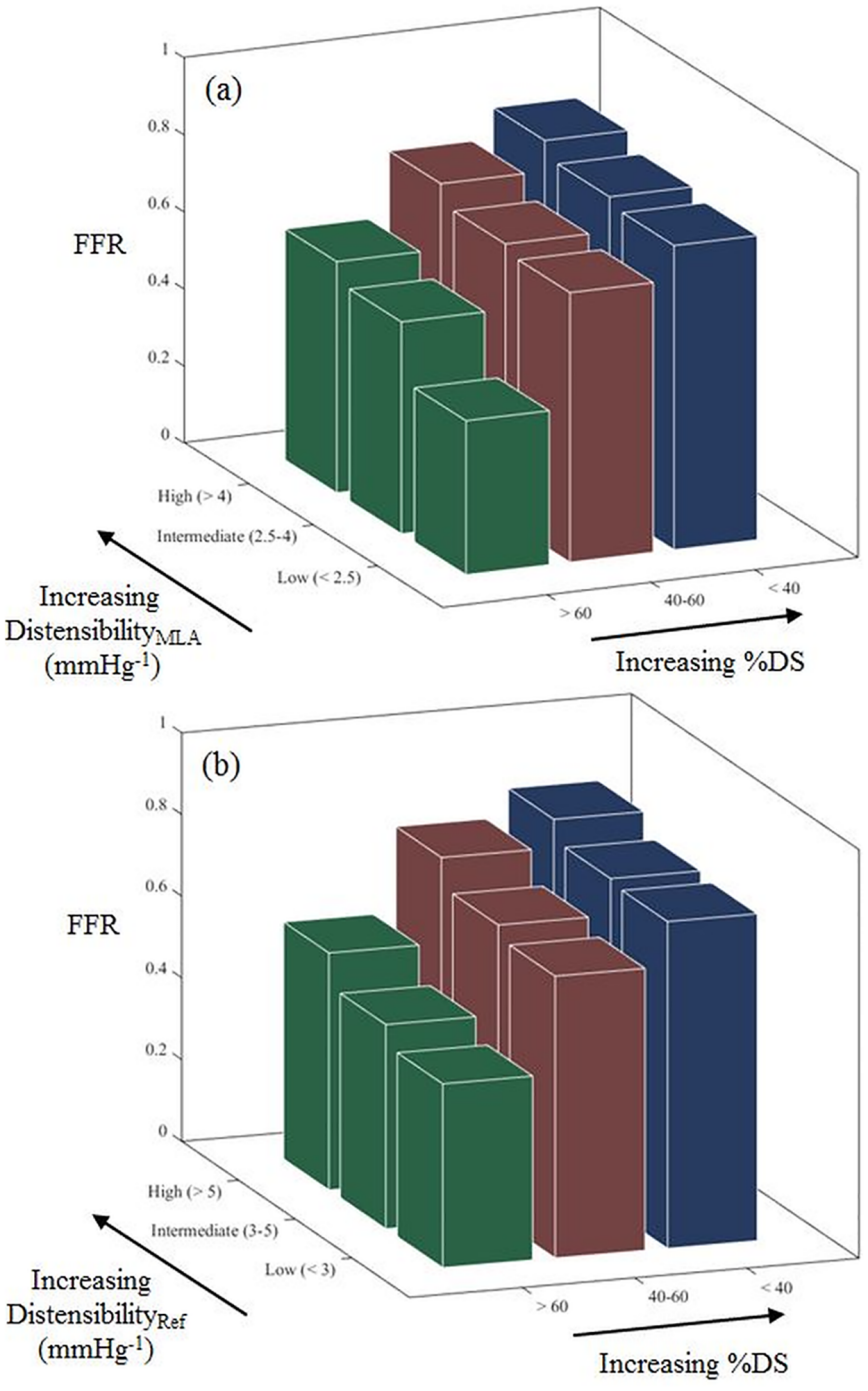
Relationship between FFR, percentage diameter stenosis and distensibility. (a) Distensibility_MLA_ and (b) Distensibility_Ref_.

FFR correlated with both Distensibility_MLA_ (r = 0.36, P < 0.001) and Distensibility_Ref_ (r = 0.44, P < 0.001) (Fig 9). Fig 10 demonstrates the inter-relationship between FFR and distensibility, and FFR and stenosis severity. When considering only patients with FFR >0.75, FFR correlated with Distensibility_MLA_ (r = 0.39, P <0.01) but not Distensibility_Ref_ (r = 0.23, P = 0.09). When Distensibility_Ref_, %DS and lesion length were included in a two-way ANCOVA analysis, there was significant interaction between Distensibility_Ref_ and %DS, and Distensibility_Ref_ was not an independent predictor of FFR (F (1, 99) = 3.09, p = 0.119). In contrast, when Distensibility_MLA_, %DS and lesion length were included in a two-way ANCOVA analysis, Distensibility_MLA_ (F (1, 100) = 4.17, p = 0.031) and %DS (F (1, 100) = 60.30, p < 0.01) were both shown to be independent predictors of FFR, and there was no interaction between Distensibility_MLA_ and %DS.

## Discussion

The results of this study demonstrate that FFR increases with increasing coronary artery distensibility at the lesion site. The effect of distensibility on FFR increases with increasing stenosis severity.

Aside from limitations in image quality, the discordance between anatomical and functional assessment of stenosis severity has been attributed to several known factors including the amount of myocardium supplied by the target vessel, lesion irregularity and length, the presence of diffuse coronary artery disease and microcirculatory impairment [3, 4]. In addition, atherosclerotic plaques are complex in shape, and plaque characteristics such as length, shape, irregularity and eccentricity will all contribute to altering the resistance to flow across a coronary lesion, and affect FFR. One previous computational study showed that increased vessel compliance, and therefore by extension distensibility, is a significant contributor to this discordance [26]. However, this study used idealized vessel geometries with area stenosis values of 70%, 80% and 90%, and did not examine the relationship of FFR and distensibility in a patient cohort. The results of our study agree with the general findings of this previous study. Moreover, we show in the clinical cohort that distensibility at the lesion site is an independent determinant of FFR whereas distensibility of the vessel distant to the lesion site was not.

The results of our study could be explained by considering the principles of fluid flow dynamics. Translesional pressure would increase in rigid vessels due to the increase in momentum change, and distensibility at the site of maximum stenosis would result in lower resistance across the lesion throughout the whole cardiac cycle. Distensibility at the site of maximum stenosis severity would therefore be more important in determining the amount of resistance to flow compared to distensibility at the non-stenosed reference vessel site. The effect of vessel distensibility on flow resistance would therefore be expected to increase with increasing stenosis severity. The range of vessel distensibility seen in our clinical cohort is very similar to that of several previously published studies [23, 24, 27–30].

FFR is an important determinant of prognosis. In patients who do not undergo revascularization, lower FFR is associated with increased adverse cardiovascular events [5, 6]. Lesions with low FFR are more likely to cause subsequent myocardial infarction [2]. The results of this study suggest that distensibility or compliance at the site of coronary lesions may contribute to the functional significance of coronary stenosis. Understanding and quantifying this process may help us understand the variations in FFR between similar angiographic stenoses.

In a large study involving patients who had CT coronary angiography, lesion calcification was found to be an independent predictor of poor prognosis [7]. This result was thought to be surprising as the conventional paradigm was that soft plaques were thought to be more “vulnerable” [31, 32]. The authors of this previous study suggested that this is due to the fact that identification of soft plaques can be difficult because of movement artifact. Our results suggest that decreased compliance at the site of coronary lesions could lead to pathogenic rheological states.

It is likely that stenosis severity and other anatomical features of the lesion interact with distensibility to determine local blood flow patterns, which in turn interact with plaque characteristics to determine plaque vulnerability. In fact, coronary distensibility was previously found to be associated with vulnerable plaque characteristics such as the presence of a necrotic core on IVUS as well as endothelial dysfunction of the epicardial vessel and microcirculation [33].

Our results also show that increased stenosis severity was associated with decreased distensibility. It is possible that vessels that are more distensible allows for greater compensatory vessel expansion in positive remodelling, leading to less severe stenosis. However, it is equally likely the decrease in distensibility in severe stenosis is due to increased plaque burden. Therefore, it remains unknown whether decreased distensibility is the cause or consequence of stenosis severity.

It was previously reported that high dose atorvastatin tends to reduce vessel stiffness [34, 35]. In our study, no correlation was found between distensibility and clinical variables. This is most likely because of the relatively small number of patients, where only highly significant variables would prove significant.

Our findings showed that lesion length correlated with Distensibility_Ref_ but not with Distensibility_MLA_. Long lesions mean that there is diffuse disease well away from the site of maximal stenosis thus decreasing Distensibility_Ref_. However, lesion length should not affect Distensibility_MLA_ because this is at the lesion site of maximal stenosis which is a single point for all arteries.

Distensibility is not easily assessed by visual interpretation of the coronary angiogram and requires specific analysis using QCA or IVUS. The results of our study support the use of FFR for lesion assessment as FFR measurement incorporates all relevant factors that determine the extent of ischaemia in relation to the epicardial stenosis such as stenosis severity, lesion length, lesion irregularity and distensibility. In considering the physiological consequence of coronary stenosis, our results demonstrate that a lesion with greater distensibility, that can now be measured with 3D-QCA, will be less functionally significant. It remains unknown whether measuring distensibility adds value to FFR in predicting adverse outcomes and will be the focus of future studies.

FFR reflects flow in the microcirculation supplied by the arterial segment being interrogated. Therefore, impaired microcirculatory flow would cause higher FFR values. However, in theory, the FFR should still reflect the functional significance of epicardial stenosis in that setting. For example, a severe stenosis in a left anterior descending artery may have FFR >0.8 in the presence of previous infarction in the anterior territory. This is because FFR takes into account myocardial viability and will provide an indication of the amount of increase in flow to the myocardium if the stenosis was stented. The FFR is only unreliable when there is an unstable microcirculation. For example, during acute ST-elevation myocardial infarction, there is a transient impairment of microcirculatory function that may recover over a period of days or weeks. The FFR therefore can potentially underestimate the significance of stenosis severity in the culprit artery in this setting. It is unknown whether vessel distensibility affects microcirculatory function, and this was not evaluated in our current study.

Several new CFD-based methods to calculate FFR without direct measurement have been developed including CT-derived FFR and 3D angiography “virtual” FFR [8–14]. Using CFD, these methods have incorporated known factors that cause anatomical-physiological discordance. However, recent studies show continued inaccuracy of these methods when compared to invasive FFR [14, 15], and these methods currently do not incorporate vessel distensibility into their simulations. The current study demonstrates that FFR may be underestimated when performing CFD analysis using rigid models, and suggests that incorporating distensibility may improve the accuracy of CFD-based methods to calculate FFR.

### Study limitations

This study has several limitations. Firstly, only patients who underwent FFR interrogation for clinical reasons were included in this study. Therefore, the relationship between vessel distensibility and FFR was only evaluated in arteries with intermediate stenosis. However, the clinical application of FFR to guide revascularization decisions is most relevant in the setting of intermediate coronary stenosis. Secondly, although vessel distensibility was directly measured, the underlying mechanism for the variation in vessel compliance could not be determined in this study and requires other types of analysis such as virtual histology IVUS. Thirdly, we have not measured flow. Although the effect of distensibility on FFR is likely due to the dynamic change in stenosis severity leading to change in resistance across the lesion site, we are uncertain whether vessel distensibility causes a change in overall flow in the target territory. Fourthly, the instantaneous wave-free ratio (iFR), which is a newer index to assess stenosis severity was not measured in this study, and it would have been of interest to investigate the relationship between iFR and distensibility. Lastly, results from the *in silico* component of this study demonstrate that distensibility is a significant determinant of FFR when microcirculatory function is kept constant. However, this condition doesn’t necessarily match the clinical situation where hyperaemia is induced in a pulsatile circulation.

## Conclusions

Coronary arterial distensibility, especially at the site of coronary stenosis, is an independent determinant of FFR, and another important contributor to the discordance between anatomical and functional assessments of stenosis severity. Techniques to indirectly calculate FFR may be improved by incorporating vessel distensibility. The value of measuring distensibility to add prognostic value to FFR warrants further investigation.

## Supporting information

S1 Table (A) Correlation between distensibility and continues variables, (B) Correlation between distensibility and dichotomous variables, (C) Correlation between distensibility and target vessel. (DOCX)

S2 Table (A) Correlation between FFR and continues variables, (B) Correlation between FFR and dichotomous variables, (C) Correlation between FFR and target vessel. (DOCX)
