# Supplementary material for: The relationship between coronary artery distensibility and fractional flow reserve
Source: PLoS One. 2017 Jul 25;12(7):e0181824. doi: 10.1371/journal.pone.0181824 (PMC5526528; doi:10.1371/journal.pone.0181824)
Supplement: S1 Table — (DOCX) [file pone.0181824.s001.docx]

**S1 Table. (A) Correlation between distensibility and continues variables**

| **Variable** | **Mean (SD)** | **Distensibility_MLA_** | **Distensibility_Ref_** |
| --- | --- | --- | --- |
| Age (yrs) | 63±9.9 | r = -0.30, P=0.0025 | r = -0.29, P = 0.0026 |
| Reference Vessel Size (mm) | 2.8 ± 0.5 | P = 0.13 | P = 0.15 |

**S1 Table. (B) Correlation between distensibility and dichotomous variables**

| **Variable** | **Distensibility_MLA_** | | | **Distensibility_Ref_** | | |
| --- | --- | --- | --- | --- | --- | --- |
|  | **Yes** | **No** | **P** | **Yes** | **No** | **P** |
| Gender (M) | 2.97±1.31 | 2.86±1.17 | 0.72 | 3.84±1.48 | 3.81±1.36 | 0.92 |
| Hypertension | 2.80±1.15 | 3.25±1.41 | 0.08 | 3.75±1.34 | 3.98±1.63 | 0.43 |
| Dyslipidaemia | 2.88±1.24 | 3.20±1.33 | 0.25 | 3.78±1.43 | 3.98±1.51 | 0.53 |
| Diabetes | 2.91±1.29 | 2.99±1.26 | 0.74 | 3.69±1.51 | 3.92±1.42 | 0.44 |
| Smoking | 3.10±1.22 | 2.88±1.30 | 0.41 | 3.99±1.31 | 3.72±1.54 | 0.31 |
| Aspirin | 2.98±1.28 | 2.70±0.73 | 0.71 | 3.87±1.46 | 3.68±0.93 | 0.79 |
| Clopidogrel | 2.97±1.35 | 2.98±1.04 | 0.99 | 3.89±1.51 | 3.81±1.26 | 0.82 |
| Beta-blocker | 3.00±1.32 | 2.94±1.20 | 0.82 | 3.90±1.56 | 3.83±1.29 | 0.80 |
| ACE-I/ARB | 2.82±1.21 | 3.27±1.34 | 0.10 | 3.76±1.45 | 4.01±1.37 | 0.29 |
| Statin | 3.03±1.29 | 2.68±1.1 | 0.33 | 3.96±1.46 | 3.36±1.26 | 0.14 |
| Calcium blockers | 2.50±1.05 | 2.62±1.12 | 0.72 | 3.66±1.26 | 3.96±1.31 | 0.45 |
| Nitrates | 2.50±1.19 | 2.64±1.07 | 0.63 | 3.48±1.22 | 4.06±1.31 | 0.11 |

**S1 Table. (C) Correlation between distensibility and target vessel**

| **Variable** | **LAD** | **LCX** | **RCA** | **P** |
| --- | --- | --- | --- | --- |
| Distensibility_MLA_ | 2.95±1.34 | 2.46±1.40 | 3.47±1.63 | 0.33 |
| Distensibility_Ref_ | 3.91±1.57 | 3.17±1.52 | 4.16±1.74 | 0.78 |
