# Supplementary material for: The relationship between coronary artery distensibility and fractional flow reserve
Source: PLoS One. 2017 Jul 25;12(7):e0181824. doi: 10.1371/journal.pone.0181824 (PMC5526528; doi:10.1371/journal.pone.0181824)
Supplement: S2 Table — (DOCX) [file pone.0181824.s002.docx]

**S2 Table. (A) Correlation between FFR and continues variables**

| **Variable** | **Mean (SD)** | **FFR** |
| --- | --- | --- |
| Age (yrs) | 63±9.9 | P = 0.77 |
| Reference Vessel Size (mm) | 2.8 ± 0.5 | P = 0.48 |

**S2 Table. (B) Correlation between FFR and dichotomous variables**

| **Variable** | **FFR** | | |
| --- | --- | --- | --- |
|  | **Yes** | **No** | **P** |
| Gender (M) | 0.66±0.19 | 0.74±0.16 | 0.12 |
| Hypertension | 0.67±0.18 | 0.68±0.19 | 0.78 |
| Dyslipidaemia | 0.68±0.18 | 0.67±0.20 | 0.81 |
| Diabetes | 0.67±0.18 | 0.68±0.18 | 0.71 |
| Smoking | 0.67±0.18 | 0.68±0.19 | 0.77 |
| Aspirin | 0.67±0.19 | 0.69±0.32 | 0.85 |
| Clopidogrel | 0.66±0.19 | 0.70±0.18 | 0.43 |
| Beta-blocker | 0.67±0.20 | 0.68±0.16 | 0.64 |
| ACE-I/ARB | 0.68±0.18 | 0.65±0.19 | 0.41 |
| Statin | 0.68±0.19 | 0.65±0.18 | 0.56 |
| Calcium blockers | 0.66±0.18 | 0.61±0.18 | 0.38 |
| Nitrates | 0.64±0.18 | 0.60±0.17 | 0.28 |

**S2 Table. (C) Correlation between FFR and target vessel**

| **Variable** | **LAD** | **LCX** | **RCA** | **P** |
| --- | --- | --- | --- | --- |
| FFR | 0.68±0.19 | 0.67±0.25 | 0.74±0.16 | 0.57 |
